# pH-Dependent Release of Vancomycin from Modularly Assembled Collagen Laminates

**DOI:** 10.3390/polym14235227

**Published:** 2022-12-01

**Authors:** Michelle Fiona Kilb, Ulrike Ritz, Daniela Nickel, Katja Schmitz

**Affiliations:** 1 Clemens-Schöpf-Institute of Organic Chemistry and Biochemistry, Technical University of Darmstadt, Alarich-Weiss-Straße 8, 64287 Darmstadt, Germany; 2 Department of Orthopaedics and Traumatology, BiomaTiCS, University Medical Center, Johannes Gutenberg University, Langenbeckstraße 1, 55131 Mainz, Germany; 3 Berufsakademie Sachsen–Staatliche Studienakademie Glauchau, University of Cooperative Education, Kopernikusstraße 51, 08371 Glauchau, Germany

**Keywords:** collagen, composite material, controlled release, vancomycin, swelling, pH dependence, rose bengal, crosslinking

## Abstract

To prevent surgical site infections, antibiotics can be released from carriers made of biomaterials, such as collagen, that support the healing process and are slowly degraded in the body. In our labs we have developed collagen laminates that can be easily assembled and bonded on-site, according to medical needs. As shown previously, the asymmetric assembly leads to different release rates at the major faces of the laminate. Since the pH changes during the wound healing and infection, we further examined the effect of an acidic and alkaline pH, in comparison to pH 7.4 on the release of vancomycin from different collagen samples. For this purpose, we used an additively manufactured sample holder and quantified the release by HPLC. Our results show that the pH value does not have any influence on the total amount of released vancomycin (atelocollagen sponge pH 5.5: 71 ± 2%, pH 7.4: 68 ± 8%, pH 8.5: 74 ± 3%, bilayer laminate pH 5.5: 61 ± 6%, pH 7.4: 69 ± 4% and pH 8.5: 67 ± 3%) but on the time for half-maximal release. At an acidic pH of 5.5, the swelling of the atelocollagen sponge is largely increased, leading to a 2–3 h retarded release, compared to the physiological pH. No changes in swelling were observed at the basic pH and the compound release was 1–2 h delayed. These effects need to be considered when choosing the materials for the laminate assembly.

## 1. Introduction

Surgical site infections (SSIs) are a common complication in trauma medicine that prolong recovery times and deteriorate the clinical outcome [1,2]. To prevent such infections, antibiotics can be administered systemically or locally. A local application avoids some side effects resulting from the systemic application. However, high local concentrations of antibiotics can interfere with the healing processes [3], so that a temporally and spatially controlled release is desirable. In particular, the controlled release from biomaterials that can remain inside the body are of interest, as the carrier does not have to be removed [4] and the appropriate drug amount can be directly applied [5]. 

Biocompatible materials that release drugs with an initial burst, followed by a slow release of smaller amounts to sustain the local concentrations are convenient. Different delivery systems with tunable properties for wound healing drugs have been developed, ranging from microspheres and nanoparticles to injectable hydrogels [4]. Zirak et al. developed porous ceramic microspheres coated with poly(lactic-co glycolic acid) (PLGA) that allow a controlled release of vancomycin and are cell compatible [6]. Microspheres without coating showed an in vitro burst release that can be explained by dominating the bioresorption while coating with the PLGA lead to a dominating effect of diffusion [6]. Somu et al. used self-assembled lysozyme nanoparticles conjugated with curcumin as a drug delivery system, in which the carrier itself is antimicrobially active [7]. Nutan et al. demonstrated that the release of the model drug 5-fluorouracil from the amphiphilic co-network gels and hydrogels, as well as their degradation, can be controlled by gel composition [8] and Singh Chandel et al. developed hydrogels, based on alkylated dextran copolymers that are suitable for the release of the hydrophobic antimycotic griseofulvin and the hydrophilic antibiotic ornidazole alike [9]. Some of these materials even exhibit regenerative properties that can be used for wound healing. Singh Chandel et al. developed water-based injectable hydrogels, based on poly[(2-dimethylamino)ethylmethacrylate]-b-poly(N-isopropyl acrylamide) and poly(ethylene glycol) that enable the proliferation of cells, the platelet adhesion and the adsorption of fibrinogen to support wound healing [10]. 

Among the biocompatible materials, the biodegradable polymers that lead to the non-toxic degradation products are particularly attractive. Poly(lactic acid) (PLA) and poly(glycolic acid) (PGA) and copolymers thereof (PLGA) belong to the most frequently used polymers in the compound delivery since they are degraded to metabolites that can be further processed in the body [11]. Commonly used biodegradable biomaterials are the polysaccharides hyaluronic acid and chitosan, as well as the protein collagen [12]. Collagen has been used for the sustained release for decades as it is inexpensive, easy to handle and its degradation products are non-toxic and non-immunogenic [12]. Furthermore, it can stimulate bone recovery by promoting the proliferation and differentiation of mesenchymal stem cells [13,14]. In previous studies, we assembled commercially available collagen sheets to collagen laminates by the photochemical crosslinking with rose bengal (RB) and green light (RGX) [15,16,17]. This convenient and inexpensive method has already been used for different clinical applications [18,19]. Using an additively manufactured sample holder we could show that the direction and time course of the antibiotic release at pH 7.4 can be controlled by the way such laminates are loaded and assembled [16]. The release studies were carried out with the model antibiotic vancomycin which is a frequently used antibiotic to prevent SSIs [1].

Healing wounds are characterized by the dynamic changes of the physiological environment [5]. The pH values observed during healing and infection can differ significantly from the physiological pH. The stages of the physiological cutaneous wound healing are characterized by the pH values ranging from pH 9.0 to 5.5 within 14 days post operation [20]. A decrease of the pH value has been reported for the cultivation of *Staphylococcus aureus* on rat jaw bones [21] and in osteotomy hematoma within 4 h after bone injury, where a pH value of 6.62 ± 0.33 was measured [22]. As the pH changes during wound healing and infection, we were interested in the effect of the pH on the direction and duration of the vancomycin release from the collagen laminates. For this purpose, we analyzed the release of vancomycin from commercially available sponge-like “Atelocollagen” and a two-layer laminate composed of Atelocollagen and a thin film of “Collagen Solutions” (AC-laminate) under acidic conditions and alkaline conditions. To adhere to the physiologically relevant pH range, a pH of 5.5 was chosen as a commonly used pH value to simulate the acidic conditions at sites of bacterial infection [23,24] and a basic pH of 8.5 was used, which is representative for the initial mean value of wounds after second degree burns [25]. 

## 2. Materials and Methods

### 2.1. Fabrication of the Collagen Samples

Collagen laminates were composed of two different commercially available collagen sheets that had already been characterized before [17]. “Atelocollagen” refers to the first type of collagen, which is an atelocollagen sponge (CLS-01) fabricated from bovine dermal type I collagen from Koken Co., Ltd. (Tokyo, Japan). “Collagen Solutions” describes a non-perforated film of bovine type I collagen from the tendon purchased from Collagen Solutions (London, UK). All buffer components were purchased from Carl Roth GmbH + Co. KG (Karlsruhe, Germany).

The fabrication of the collagen laminates using RGX was performed as described by Kilb et al. [16]. In detail, the collagen sheets with a size of 1 cm × 1 cm were loaded with 0.01% (*w*/*v*) RB (Alfa Aesar, Haverhill, MA, USA) in a phosphate buffered saline (PBS, 137 mM sodium chloride, 2.7 mM potassium chloride, 1.5 mM potassium dihydrogen phosphate, 8.1 mM disodium hydrogen phosphate, pH 7.4) in the dark at room temperature (RT). The loading was carried out by swelling the sheets for 2 h in a Petri dish (SARSTEDT, Nümbrecht, Germany) in a total volume that fits the loading capacity. The latter was determined as the weight difference of a dry collagen sample and of the sample after swelling with PBS [17]. For the fabrication of AC-laminates, the loaded Atelocollagen and Collagen Solutions sheets were placed onto each other with 20 µL of 0.01% (*w*/*v*) RB solution in PBS in-between. The stacked sheets were crosslinked to each other by exposure to green light (λ = 565 nm, M565L3, Thorlabs GmbH, Bergkirchen, Germany) for 10 min.

Single RGX-modified collagen sheets were similarly prepared and directly exposed to green light after loading with 0.01% (*w*/*v*) RB in PBS.

### 2.2. Release of Vancomycin

The release of vancomycin was determined, as described by Kilb et al. [16]. All buffer components were purchased from Carl Roth GmbH + Co. KG (Karlsruhe, Germany). 

For the release of vancomycin from the crosslinked Atelocollagen and AC-laminates, the collagen samples were prepared, as described in Section 2.1 with the modification that the 0.01% (*w*/*v*) RB solution in PBS used for the loading of Atelocollagen was supplemented with vancomycin hydrochloride (Carl Roth GmbH + Co. KG, Karlsruhe, Germany), corresponding to an uptake of 1 mg vancomycin. The preparation of the Collagen Solutions sheet for the laminate was performed as described in Section 2.1. The respective collagen sample was placed between both parts of the sample holder that was fabricated from a standard white resin V4 (Formlabs, Somerville, MA, USA) [16]. The sample holder was placed into a cavity of a non-treated 24-well tissue culture plate (VWR International, Radnor, PA, USA) that was filled with 1 mL of PBS, MES buffer (140 mM sodium chloride, 9.6 mM MES, pH 5.5) or Tris buffer (140 mM sodium chloride, 9.6 mM Tris base, pH 8.5). In the upper part of the sample holder, 1 mL of PBS, MES buffer or Tris buffer was added as well. The incubation was performed at 37 °C and the liquid from the upper and lower parts of the sample holder was completely withdrawn at different time points. In the next step, 1 mL of fresh buffer was added to both cavities of the sample holder. The vancomycin content was analyzed by the reversed-phase HPLC, as described by Eckes et al. [17] and Kilb et al. [16]. A detailed description can be found in the Supplementary Materials. 

### 2.3. Determination of the Swelling Degree of Collagen Samples

The swelling degree of the collagen laminates and the single RGX-modified collagen sheets was determined by a method reported previously by Eckes et al. and Braun et al. [15,17]. In brief, the collagen samples were prepared, as described in Section 2.1. The samples were freeze-dried overnight and the dry weight (m_dry_) of each sample was determined in triplicates. Each collagen sample was transferred to a well of a non-treated, flat bottom 24-well microplate (VWR, Radnor, PA, USA) and incubated for 2 h in 2 mL of the appropriate buffer (PBS pH 7.4, MES pH 5.5 or Tris pH 8.5) at 37 °C. The non-absorbed liquid was removed from the samples by carefully blotting the samples on green paper towels and the wet weight (m_wet_) of the samples was determined in triplicates. The swelling degree was calculated from the triplicates, according to equation 1. The significance levels of the differences between the experimentally determined mean swelling degrees were analyzed by a Kruskal–Wallis–ANOVA at *p* = 0.05 with OriginPro 2021b (OriginLab Corporation, Northampton, MA, USA).

Swelling degree [%] = ((m_wet_−m_dry_)/m_dry_) × 100% (1)

## 3. Results

### 3.1. Release of Vancomycin from the RGX-Modified Atelocollagen

**Figure 1:**
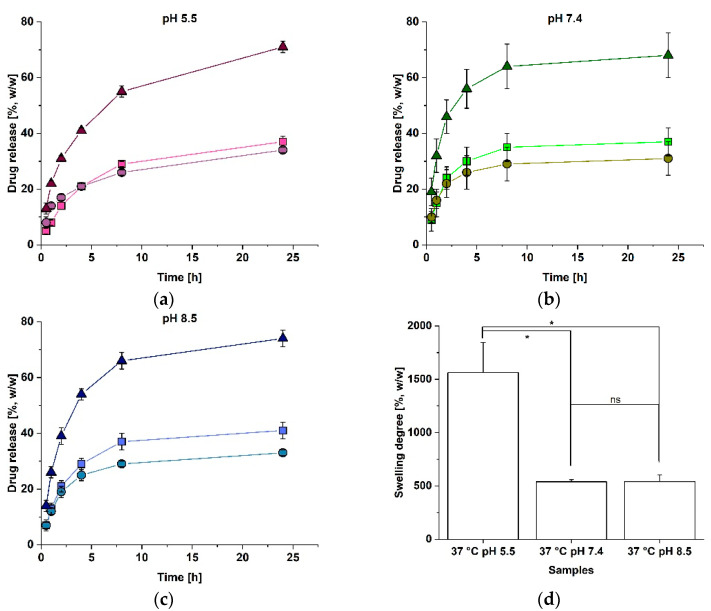
Vancomycin release and swelling properties of the RGX-modified Atelocollagen at different pH values. The release of vancomycin was determined over 24 h in the upper chamber (squares), lower chamber (dots) and in total (triangles). The amount of released vancomycin was calculated according to the amount (1 mg) loaded into the Atelocollagen layer. Error bars represent the standard deviation (n = 3); (**a**) Release at pH 5.5; (**b**) Release at pH 7.4 (data obtained from Kilb et al. [16]); (**c**) Release at pH 8.5; (**d**) Swelling degree after 2 h of incubation at 37 °C at different pH values. A Kruskal–Wallis–ANOVA was used to determine the significance of the difference of the mean swelling degrees at *p* ≤ 0.05, indicated by an asterisk. Non-significant differences are indicated by ns.

As the pH value changes during the physiological cutaneous wound healing from pH 9.0 to pH 5.5 and the microbial contamination also leads to a moderately acidic environment at the sites of infection [20,23,24], the release of vancomycin from a single RGX-modified sheet of Atelocollagen was examined at pH 5.5 and pH 8.5. The release was compared to the release data at pH 7.4, that has been published previously [16]. As shown in Figure 1a–c, the total amount of released vancomycin after 24 h of incubation did not differ between pH 5.5, pH 7.4 and pH 8.5 (pH 5.5: 71 ± 2%, pH 7.4: 68 ± 8% [16] and pH 8.5: 74 ± 3%). For pH 5.5, the time for the half-maximal release was reached three hours later than at pH 7.4 and for pH 8.5, the time for the half-maximal release was reached one hour later than under the physiological pH (pH 5.5: 41 ± 1% (4 h), pH 7.4: 32 ± 6% (1 h) [16] and pH 8.5: 39 ± 3% (2 h)). At pH 5.5, equal amounts of vancomycin were released into the upper and the lower cavity, similar to the release results at the physiological pH [16]. At pH 8.5, only slightly more vancomycin was detected in the upper cavity after 8 h.

To analyze whether the elongated times for the half-maximal release at pH 5.5 and pH 8.5 can be explained by the pH-dependent swelling, the swelling degrees of the RGX-modified Atelocollagen at pH 5.5, pH 7.4 and pH 8.5 were examined and compared. The result in Figure 1d shows that the mean swelling degrees differed significantly between pH 7.4 and pH 5.5, as a 65% lower swelling degree was reached at pH 7.4. No significant difference was found between pH 7.4 and pH 8.5 (pH 5.5: 1562 ± 283%, pH 7.4: 539 ± 20% and pH 8.5: 541 ± 64%). 

### 3.2. Release of Vancomycin from the Two-Layer Heterogenous Laminates (AC-Laminates)

**Figure 2:**
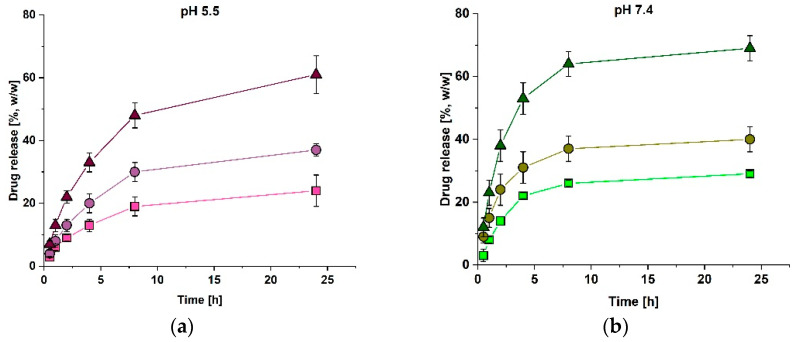

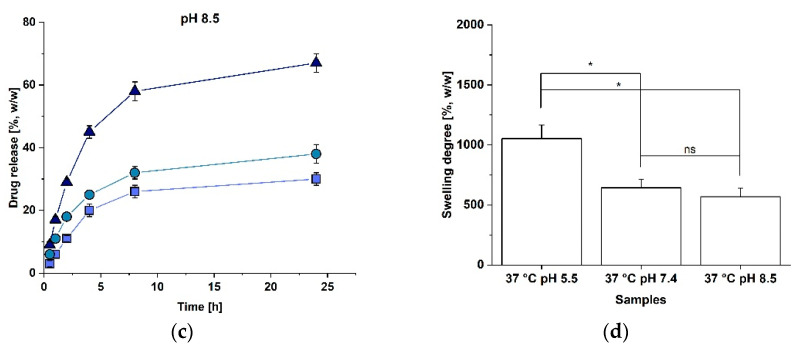
Vancomycin release and the swelling properties of the AC-laminates at different pH values. The release of vancomycin was determined over 24 h in the upper chamber (squares), the lower chamber (dots) and in total (triangles). The amount of the released vancomycin was calculated according to the amount (1 mg) loaded into the Atelocollagen layer. This layer of the AC-laminate was facing the upper chamber of the sample holder. Error bars represent the standard deviation (n = 3); (**a**) Release at pH 5.5; (**b**) Release at pH 7.4 (data obtained from Kilb et al. [16]); (**c**) Release at pH 8.5; (**d**) Swelling degree after 2 h of incubation at 37 °C at different pH values. A Kruskal–Wallis–ANOVA was used to determine the significance of the difference of the mean swelling degrees at *p* ≤ 0.05, indicated by an asterisk. Non-significant differences are indicated by ns.

To study the release from the asymmetric bilayer-laminates, the AC-laminates were examined that had already shown a collagen-sheet-dependent release of vancomycin at pH 7.4. [16] As higher amounts of vancomycin were released, if antibiotics had been loaded into the Atelocollagen layer, [16] this setup was also chosen to study the release at other pH values. The laminate was positioned in the sample holder with Atelocollagen facing the upper chamber, as previous experiments had shown that this setup facilitated the handling and that an unequal release is independent from the orientation of the laminate in the sample holder [16]. Release studies were performed at pH 5.5 and pH 8.5 and compared to the published release data at pH 7.4 [16]. As shown in Figure 2a–c, the amounts of the totally released vancomycin reached similar amounts after 24 h under all conditions (pH 5.5: 61 ± 6%, pH 7.4: 69 ± 4% [16] and pH 8.5: 67 ± 3%). Compared to the physiological pH, the time for the half-maximal release was reached two hours later at pH 5.5 and pH 8.5 (pH 5.5: 33 ± 3% (4 h), pH 7.4: 38 ± 5% (2 h) [16] and pH 8.5: 45 ± 2% (4 h)). Similar to the findings at pH 7.4 [16], more vancomycin was released at the surface of Collagen Solutions at pH 5.5 and pH 8.5, even though vancomycin was loaded into Atelocollagen. 

The swelling analysis was performed to analyze whether the elongated times for the half-maximal release at pH 5.5 and pH 8.5 might be explained by the pH-dependent differences in the swelling. As for the single Atelocollagen sheets, the swelling degrees at pH 5.5 and pH 7.4 differed significantly, since the swelling at pH 7.4 was about 39% lower than at pH 5.5. At pH 8.5, the AC-laminate reached a similar swelling degree as at pH 7.4 and the swelling degree significantly differed from pH 5.5 (pH 5.5: 1053 ± 114%, pH 7.4: 643 ± 69% and pH 8.5: 568 ± 73%). As the swelling degree of a single RGX-modified sheet of Atelocollagen was significantly higher than a single sheet of the RGX-modified Collagen Solutions (see Figure S2), the swelling effect of the Collagen Solutions layer in the AC-laminate can be neglected.

## 4. Discussion

As the pH value changes during the wound healing [20] and might have an influence on the release of antibiotics and other drugs from the collagen materials, the release of vancomycin from the RGX-modified Atelocollagen and a modularly assembled collagen laminate composed of Atelocollagen and Collagen Solutions was examined under acidic and alkaline conditions. The results are summarized in Table 1.

The total maximum releases were similar under all tested conditions, indicating that the total amounts of released vancomycin from both samples are not influenced by the pH value.

Noticeably, the swelling at pH 5.5 was significantly enhanced, compared to pH 7.4 and pH 8.5. During the swelling, an osmotic pressure gradient across the solvent-polymer interface leads to the uptake of fluid [26]. The resulting swelling is limited by the polymeric network structure [27] and at an equilibrium state, the osmotic force equals the elastic force of the network contracting in the opposite direction and thereby limiting the swelling [28]. The minimum swelling takes place at the isoelectric point [26]. For collagen, the pH-dependent swelling has already been reported in the literature [29]. As the isoelectric point of Atelocollagen lies close to pH 8 [30], the polymer bears a positive net charge at a pH of 5.5, resulting in a repulsion of the electric charges, which permits the biopolymer fibers to stretch out longer so that the swelling degree increases, in contrast to pH 7.4 and pH 8.5 [29]. Since pH 7.4 and pH 8.5 lie near the isoelectric point of Atelocollagen, the net charge is low and the swelling behavior does not differ between pH 7.4 and pH 8.5, as the data confirm.

At a pH of 5.5, the times for the half-maximal release were two to four times longer, compared to pH 7.4. As the swelling degrees for both samples at pH 5.5 were significantly larger than at pH 7.4, the elongated times for the half-maximal release might be explained by the different swelling properties of the samples at an acidic pH value. We have previously assumed that the influx of the buffer during the swelling retards the release of vancomycin [16] so that an increased swelling degree and therefore increased fluid influx leads to elongated times for the half-maximal release. The same effect also accounts for the retarded release at the Atelocollagen side of an AC-laminate that has been observed at pH 7.4 [16]. As expected, at pH 5.5, with increased swelling of Atelocollagen, this effect is more pronounced. For the AC-laminate, the swelling effect of Atelocollagen dominates, compared to the swelling of Collagen Solutions (see Figure S2). The release of equal amounts of vancomycin into both directions for Atelocollagen at pH 5.5 meets the expectations as both release areas can be considered as equal to each other [16]. 

At a pH of 8.5, the samples of RGX-modified Atelocollagen and the AC-laminate showed an elongated time for the half-maximal release, compared to pH 7.4. These findings cannot be explained by swelling, since the swelling degrees did not differ between pH 8.5 and pH 7.4 for either sample. Compared to pH 5.5, the times for the half-maximal release were either equal or shorter. This might be explained by electrostatic interactions between vancomycin and collagen, since vancomycin is negatively charged under alkaline conditions [31] and might interact with the remaining positively charged areas of the collagen chains. In the literature, strong electrostatic interactions between negatively charged rose bengal and positively charged amino groups of a collagen-like peptide have already been reported [32]. At pH 5.5, vancomycin is positively charged and should be repelled by the positive net charge of the biopolymer. However, this may be counteracted by the stronger effect of swelling.

In light of these findings, Atelocollagen should rather be omitted for antibiotic delivery if signs of an infection are already eminent as this would slow down the antibiotic release. A (collagen) material with a pH between 5 and 6 and a more rigid structure with less swelling propensity would be preferable. Moreover, the retarded release of drugs to support wound or bone healing, while infection lasts and pH is lower, could be desirable, e.g., for the therapeutically used growth factors. 

## 5. Conclusions and Outlook

As the pH value changes during the wound healing as well as infection [20], the release of vancomycin from the RGX-modified atelocollagen sponge and a modularly assembled laminate composed of Atelocollagen and Collagen Solutions were analyzed. The total amount of vancomycin released over 24 h was independent of pH (atelocollagen sponge pH 5.5: 71 ± 2%, pH 7.4: 68 ± 8%, pH 8.5: 74 ± 3%, bilayer laminate pH 5.5: 61 ± 6%, pH 7.4: 69 ± 4% and pH 8.5: 67 ± 3%). At pH 5.5, the swelling of the atelocollagen sponge was increased, leading to an elongated time for half-maximal release, compared to the physiological pH. At pH 8.5, where the swelling was comparable to pH 7.4, the time for half-maximal release was also elongated, compared to pH 7.4, which may be explained by the electrostatic interactions between vancomycin and collagen. The latter might be counteracted by the strong swelling at pH 5.5. Thus, when choosing collagen materials to improve wound healing, the interplay between the swelling of collagen and the electrostatic interactions between collagen and the antibiotic that both depend on their isoelectric point, need to be considered. These effects could be used to withhold the bioactive compounds while infections—that lead to lower pH values—last. In future studies, further commercially available collagen materials should be tested, in terms of the antibiotic release at different pH values. Furthermore, the release of other biomolecules from collagen laminates, e.g., antimicrobial peptides or bone morphogenetic proteins, should be characterized. In order to understand the mechanism of the drug release from collagen laminates and to enable the customized design of collagen laminates for drug delivery, the release of vancomycin from different collagen laminates at different conditions will be modelled mathematically, in future studies. 

## 6. Patents

Schmitz, K.; Ritz, U.; Nickel, D.; Oechsner, M.; Beyrich, T.; Rommens, P.M. ”Antimikrobielle, gewebsregenerierende Laminate für die regenerative Medizin” (DE102017126149_A1).

## Figures and Tables

**Table 1:** Overview of the results. The data indicated by the asterisk refers to a previous publication by Kilb et al. and was added to the table for comparison [16].

Laminate or Single Sheet		Released Vancomycin		Swelling Degree	Release Direction
Name	Property	After 24 h	Half-Max. Release		
A (RGX)	pH 5.5	71 ± 2%	41 ± 1% (4 h)	1562 ± 283%	Equal
A (RGX)	pH 7.4	68 ± 8% *	32 ± 6% (1 h) *	539 ± 20%	Equal *
A (RGX)	pH 8.5	74 ± 3%	39 ± 3% (2 h)	541 ± 64%	Unequal after 8 h and 24 h (upper > lower cavity)
AC	pH 5.5	61 ± 6%	33 ± 3% (4 h)	1053 ± 114%	Unequal (C > A)
AC	pH 7.4	69 ± 4% *	38 ± 5% (2 h) *	643 ± 69%	Unequal (C > A) *
AC	pH 8.5	67 ± 3%	45 ± 2% (4 h)	568 ± 73%	Unequal (C > A)

## Data Availability

The data presented in this study are available upon request from the corresponding author.

## Supplementary Materials

The following supporting information can be downloaded at: https://www.mdpi.com/article/10.3390/polym14235227/s1, Figure S1: Calibration curve of vancomycin from 0 to 1 mg/mL, Figure S2: Comparison of the swelling properties of the RGX-modified Collagen Solutions (grey) and Atelocollagen (white).
