# Supplementary material for: pH-Dependent Release of Vancomycin from Modularly Assembled Collagen Laminates
Source: Polymers (Basel). 2022 Dec 1;14(23):5227. doi: 10.3390/polym14235227 (PMC9740012; doi:10.3390/polym14235227)
Supplement: Supplementary file 1 [file polymers-14-05227-s001.zip › polymers-2028463-supplementary.pdf]

pH-dependent release of vancomycin from modularly assembled collagen laminates

Michelle Fiona Kilb [1], Ulrike Ritz [2], Daniela Nickel [3] and Katja Schmitz [1,*]

[1] Clemens-Schöpf-Institute of Organic Chemistry and Biochemistry, Technical University of Darmstadt, Alarich-Weiss-Straße 8, 64287 Darmstadt, Germany
[2] Department of Orthopaedics and Traumatology, BiomaTiCS, University Medical Center, Johannes Gutenberg University, Langenbeckstraße 1, 55131 Mainz, Germany
[3] Berufsakademie Sachsen–Staatliche Studienakademie Glauchau, University of Cooperative Education, Kopernikusstraße 51, 08371 Glauchau, Germany
* Correspondence: katja.schmitz@tu-darmstadt.de; Tel.: +49-6151-16-21015

Supplementary Material

Analysis of vancomycin release

In brief, a Shimadzu HPLC system consisting of two LC-20AD pumps, a SIL-20ACHT autosampler, a CBM-20A system controller, a SPD-M20A photodiode array detector, a DGU-403 degassing unit and a C18 Synergy™ 4 µm Fusion-RP 80 Å 250 4.6 mm column (Phenomenex Inc., Torrance, CA, USA) was used for sample analysis. 100 µL of the sample were injected onto the column and the column was washed with 100 % eluent A (95 % ultrapure water, 5 % acetonitrile, 0.1 % TFA) for 20 min at a flow rate of 0.5 mL/min. The concentration of eluent B (95 % acetonitrile, 5 % ultrapure water, 0.1 % TFA) was increased over 50 min from 0 % to 90 % at a flow rate of 0.5 mL/min. A washing step was carried out with 100 % eluent A for 10 min at a flow rate of 0.5 mL/min. Vancomycin absorption was monitored at 280 nm and collagen absorption was monitored at 220 nm. Vancomycin eluted after 40 min and the quantity of released vancomycin was calculated from a calibration curve (see Figure S1).

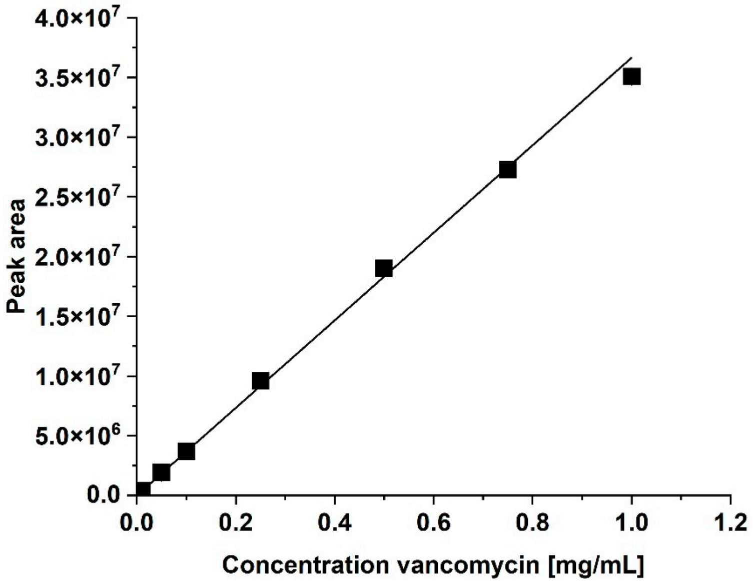

Figure S1. Calibration curve of vancomycin from 0 to 1 mg/mL. The adjusted R-square has a value of 0.9985. The intercept has a value of 248158 and the slope a value of $4x10^7$. Error bars represent the standard deviation (n=3).

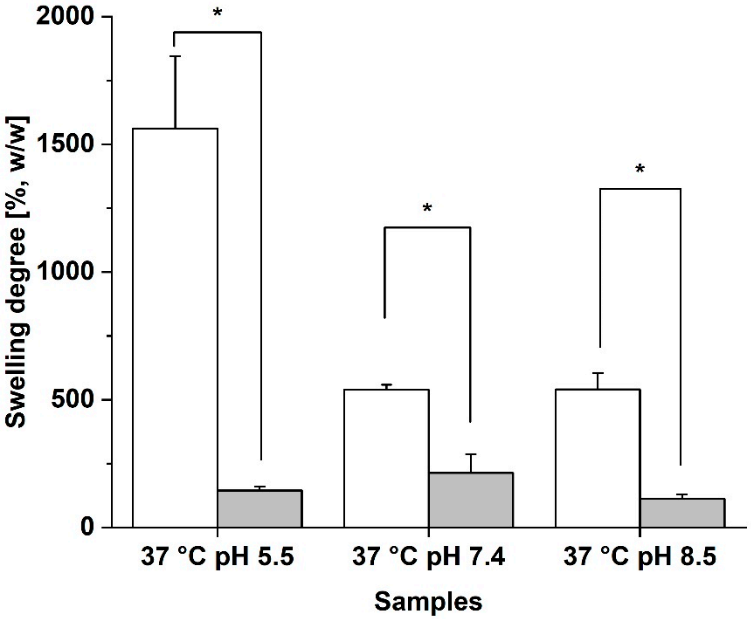

Figure S2. Comparison of the swelling properties of RGX-modified Collagen Solutions (grey) and Atelocollagen (white). Swelling analysis was performed after 2 h of incubation at 37 °C at different pH values. Error bars represent the standard deviation (n=3). Kruskal-Wallis-ANOVA was used to determine the significance of the difference of the mean swelling degrees of Atelocollagen and Collagen Solutions at $p \leq 0.05$, indicated by an asterisk.
